# Mini PCNL has gained more recognition in stone treatment guidelines

**DOI:** 10.1186/s40779-025-00606-2

**Published:** 2025-05-06

**Authors:** Ben H. Chew

**Affiliations:** The Department of Urologic Sciences, University of British Columbia, Vancouver, BC V5Z 1M9 Canada

**Keywords:** Miniaturized percutaneous nephrolithotomy (mPCNL), Minimally invasive percutaneous nephrolithotomy (mini PCNL), Nephrolithiasis, Stone

Recently, the International Alliance of Urolithiasis (IAU) released a consensus on miniaturized percutaneous nephrolithotomy (mPCNL), which was published in the *Military Medical Research* [1]. This endeavor convened an international panel of experts in mPCNL and achieved a focused consensus on this evolving technique. Considering that standard PCNL has traditionally dominated the field, establishing standardized statements through this consensus is pivotal for the global adoption and popularization of mPCNL techniques.

The transition toward miniaturization of surgery has long been a central goal for urologists. The advent of mPCNL represents a significant advancement in this regard, as studies have demonstrated that mPCNL can reduce postoperative bleeding, minimize postoperative pain, and shorten hospital stays [2–4]. Importantly, the stone-free rates achieved with mPCNL are comparable to those of standard PCNL in patients with a moderate renal stone burden (2–4 cm). This is particularly significant given the rising global prevalence of urolithiasis, thus necessitating effective and safe minimally invasive treatment options.

This IAU consensus addresses several unresolved controversies that have hindered the widespread adoption of mPCNL from previous guideline [5]. These include the various definitions of mPCNL, its comparison to standard PCNL, optimal surgical indications, perioperative and postoperative management strategies, as well as procedural tips and techniques that have yet to achieve broad consensus. By reducing these gaps, the consensus aims to establish a comprehensive clinical framework to enhance urologists’ understanding and application of mPCNL.

A lack of unified terminology has long complicated discussions surrounding mPCNL. The consensus rightly emphasizes that the term “minimally invasive PCNL (mini PCNL)” lacks precision. Instead, “miniaturized PCNL (mPCNL)” emerges as a more appropriate descriptor, encapsulating both the minimal invasiveness and the reduced size of access sheaths compared to conventional PCNL.

The issue of radiation also spans the entire patient journey, from preoperative imaging and intraoperative guidance to postoperative follow-up. Debates continue regarding the optimal imaging modalities and the use of fluoroscopy, ultrasound, or a combination of both for puncture and tract establishment during the procedure. The consensus supports all of the above modalities which have been shown to all be equal depending on surgeon comfort, skill and technique.

Furthermore, the IAU consensus highlights the procedural complexities of mPCNL, particularly regarding the retrieval of stone fragments and dust. The extraction process through the nephrostomy tract or the ureter is often the most time-consuming aspect of the procedure. The size of access channel significantly limits the maximum size of stone fragments that can pass, which directly impacts the overall operation time. This aligns with the consensus’s emphasis on adopting techniques that balance minimizing the access tract size with maximizing stone retrieval efficiency.

Despite the recent advancements, such as flexible and navigable suction ureteral access sheaths (FANS) in retrograde intrarenal surgery (RIRS), urologists remain cautious about extending the application of RIRS to treat renal stones in the range of 2–3 cm. For larger stones, mPCNL remains the first-line treatment option. However, for complete staghorn calculi or stones larger than 5 cm, operative time is significantly prolonged when mPCNL techniques are utilized [6–8]. The consensus highlights the need for appropriate surgical indications and advanced techniques to ensure the safety and efficacy of mPCNL procedure.

It is crucial to emphasize that mPCNL is not merely the same procedure through a miniature access tract. It requires a specialized skill set that must be developed alongside the adoption of these techniques. The consensus has laid out numerous procedural tips and tricks, offering urologists a valuable standardized reference. These insights are essential for mastering mPCNL and navigating the intricacies of the procedure effectively.

On another note, patient-reported outcomes are vital as they offer insights into patient’s perspective. It is concerning that only 28.1% of participants are familiar with the Wisconsin stone quality of life for evaluating quality of life in urolithiasis patients [1]. More advocacy is required to look into providing enhanced patients’ journey in the treatment of renal stones.

Consensus in medical science is imperative for establishing standards that guide clinical practice. It ensures that current standards reflect the best available knowledge, facilitate meaningful data comparisons, and promote best practices. This timely update provided by the IAU consensus will undoubtedly enhance urologists’ proficiency in mastering mPCNL techniques, ultimately highlighting the true value of mPCNL among the armamentarium of surgical options.

In conclusion, the IAU consensus on mPCNL serves as a pivotal resource for urologists, offering clear guidelines and insights to improve clinical practice and patient outcomes. As the field continues to evolve, ongoing education and adherence to these consensus recommendations will be vital for optimizing renal stone management and upholding high standards of care. Future studies comparing FANS vs. mPCNL will offer urologists further direction on how these two different technologies will be best utilized to care for patients.

## Data Availability

Not applicable.

## Abbreviations

FANS: Flexible and navigable suction ureteral access sheaths
IAU: International Alliance of Urolithiasis
mPCNL: Miniaturized percutaneous nephrolithotomy
RIRS: Retrograde intrarenal surgery
